# Supplementary material for: Ginseng-Induced Changes to Blood Vessel Dilation and the Metabolome of Rats
Source: Nutrients. 2020 Jul 27;12(8):2238. doi: 10.3390/nu12082238 (PMC7468881; doi:10.3390/nu12082238)
Supplement: Supplementary file 1 [file nutrients-12-02238-s001.pdf]

Supplementary Table 1. Ginsenosides component of ginseng extract

Ginsenoside	Amount (ppm)
Rb1	1011.5
Rb2	1124.2
Rb3	216.7
Rc	2024.9
Rd	603.1
Rg3	1615.2
Rh2	0.0
Re	941.6
Rg1	873.8
Rg2+Rh1	447.6
Rh1	26.9
Total ginsenoside	8885.5

Ginsenosides were analyzed on HPLC system (Chrompass data system, JASCO, Japan) with photodiode array detector(MD-2010, JASCO) equipped with C18 column (250x4.6 mm, SunFireTM, waters Corp, USA) with 1.6mL/min flow rate at 35℃. The mobile phase were water(solvent A) and acetronitrile(solvent B).

Supplementary Table 2. MRM mass spectrometric parameters of identified steroid hormones

	RT(min)	Metabolite	MRM mode	collision energy (eV)
**Plasma**	0.62	estradiol-3-glucuronate	473.3 > 200.9	20
	3.05	estrone-3-hemisuccinate	371.2 > 177.2	20
	9.88	11a-hydroxyestradiol	289.3 > 253.1	20
	14.53	21a-hydroxyprogesterone	331.3 > 313.0	10
**Urine**	0.63	27-hydroxycholesterol	367.3 > 114.9	10
	4.17	metandienone	301.2 > 149.2	20
	5.42	2-hydroxyestrone-1+4-N-acetylcysteine	448.3 > 161.9	20
	8.65	6-dehydroestradiol diacetate	355.3 > 313.4	20
	10.07	estriol-16,17-diacetate	373.3 > 313.2	10
	10.33	2,3-dimethoxyestradiol	317.3 >302.2	20
	14.15	5-androstenediol	273.3 > 255.1	20
	16.65	11b-hydroxyl estradiol	289.3 > 106.0	15

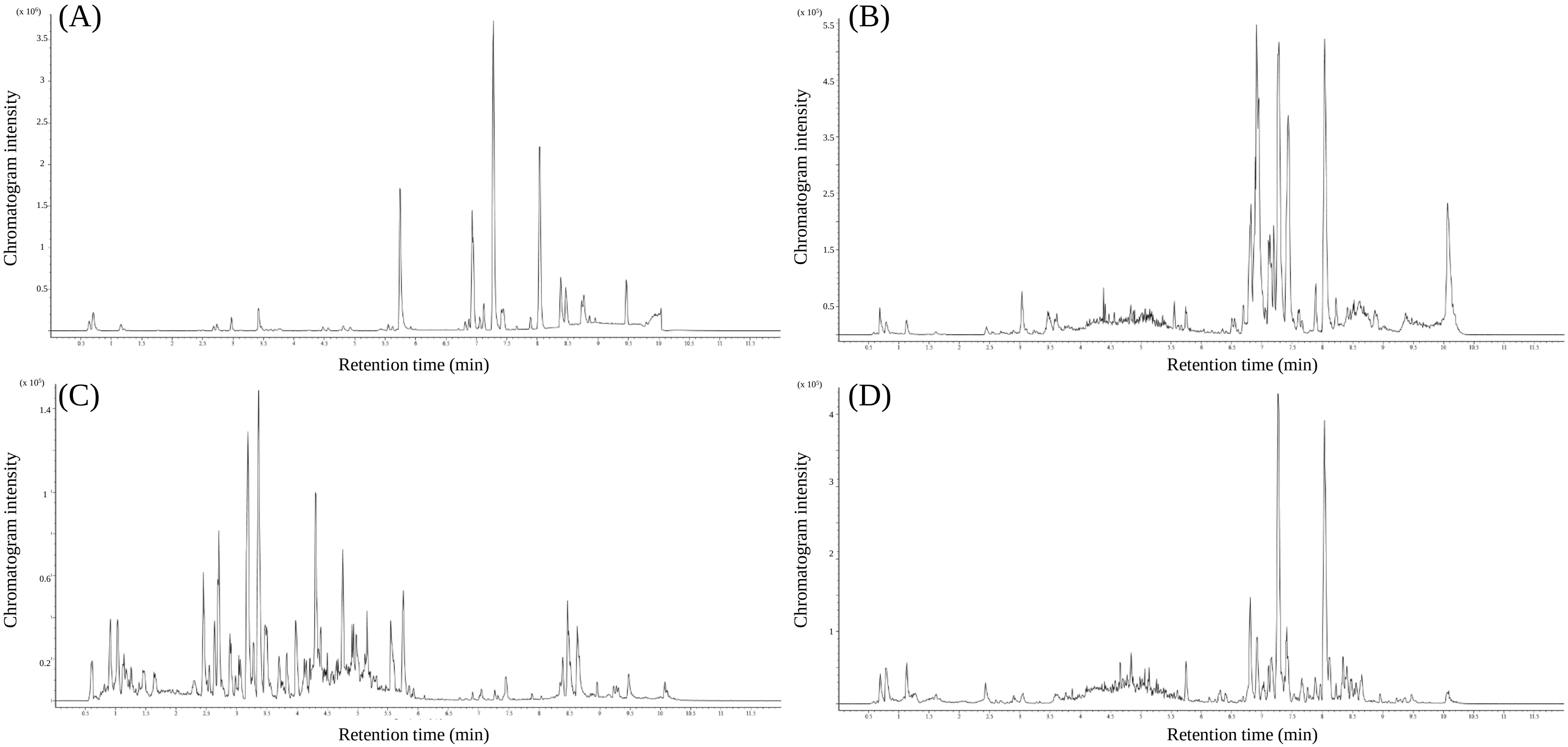

Supplementary Figure 1. Representative ultra-performance liquid chromatography-quadrupole-time-of-flight mass spectrometry (UPLC-Q-TOF MS) profiles of plasma (A), kidney (B), urine (C), and liver (D) of rats treated with oral administration of the ginseng extract.

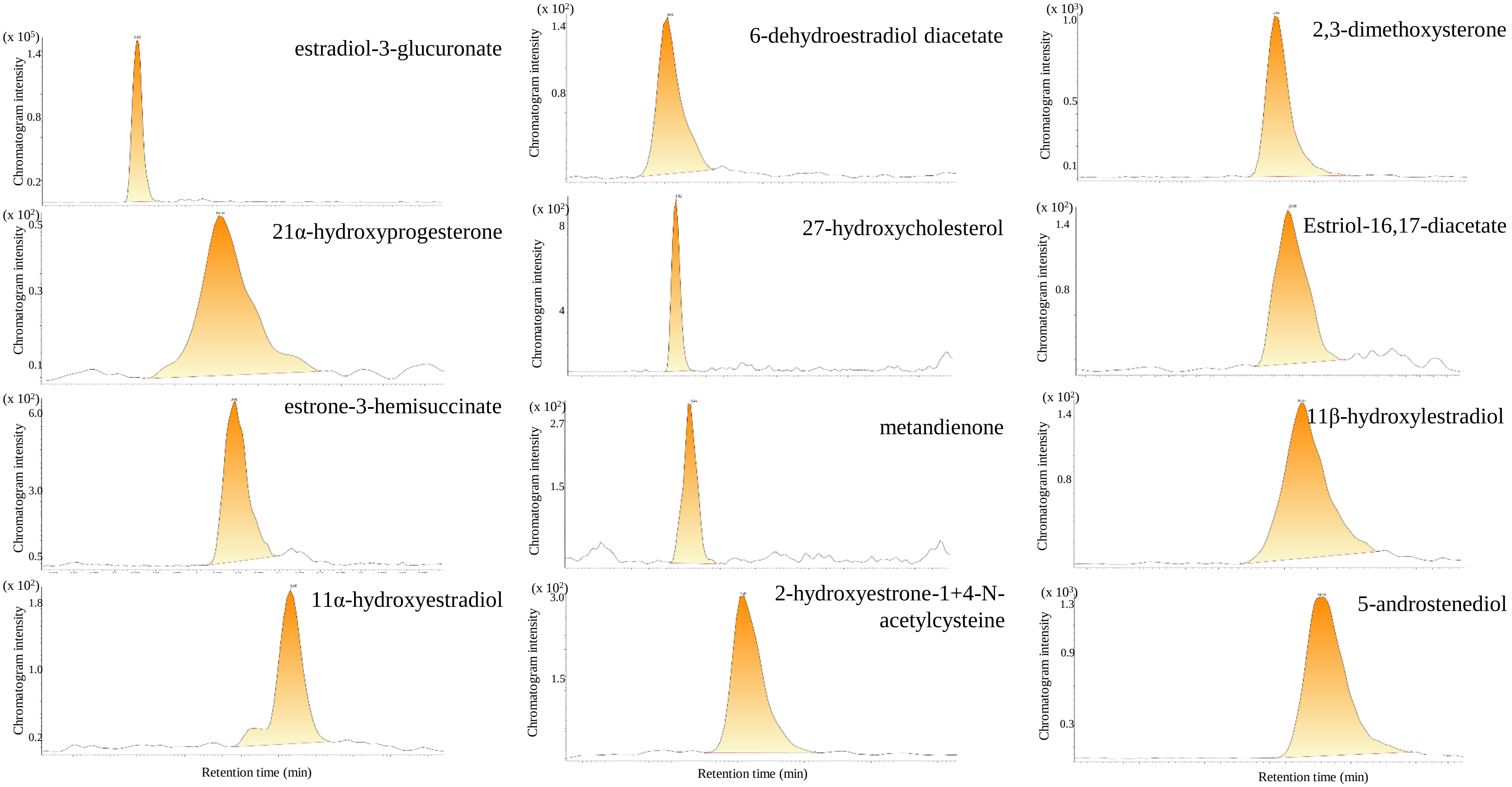

Supplementary Figure 2. Representative plasma and urinary steroid hormone chromatograms analyzed UPLC-Q-TOF MS with MRM mode.

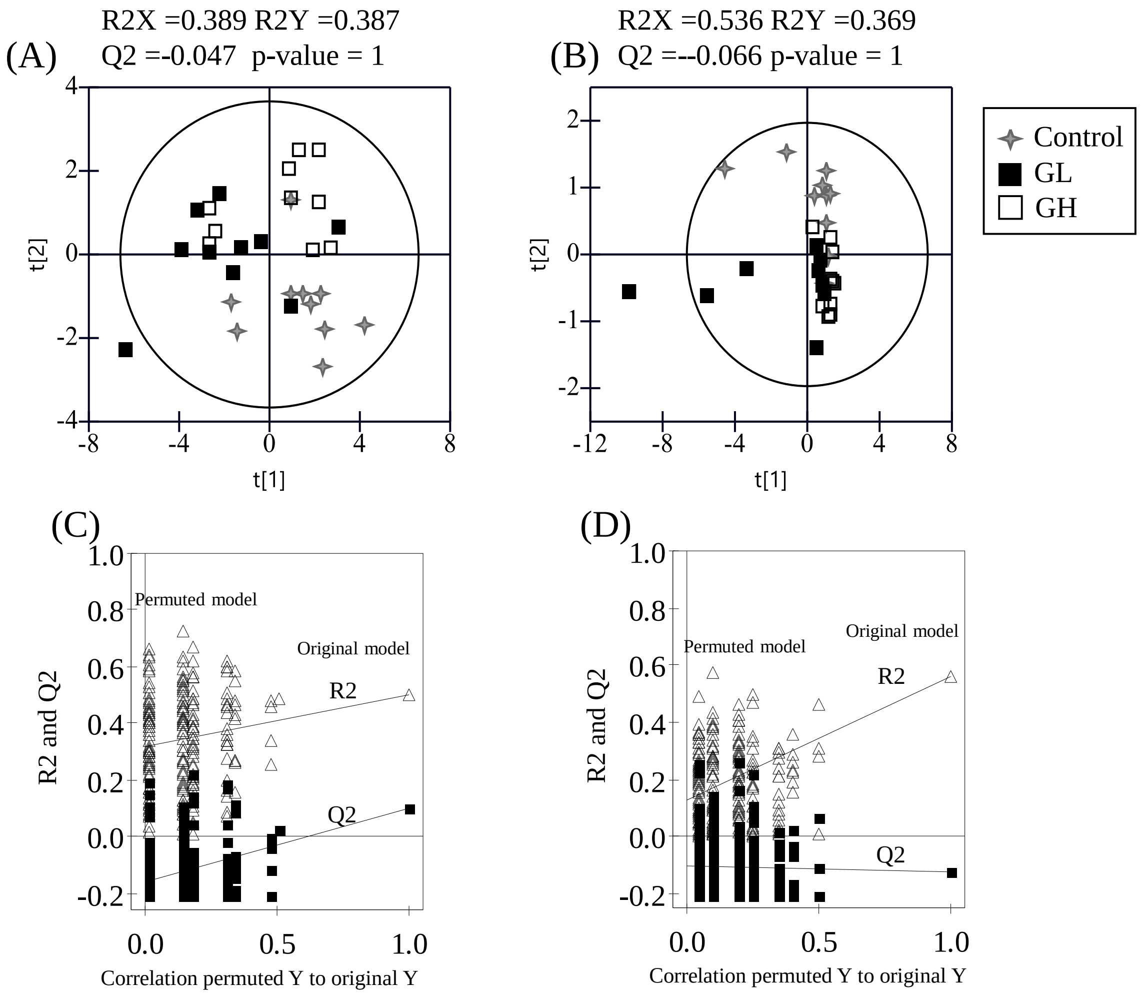

Supplementary Figure 3. Partial least-squares discriminant analysis (PLS-DA) score plots of urine (A) and liver (B) metabolites and their quality parameters. The quality for the PLS-DA scores are evaluated with R2X, R2Y, Q2, and *p*-values and validated with cross validation by 200 permutation tests of urine (C) and liver (D).

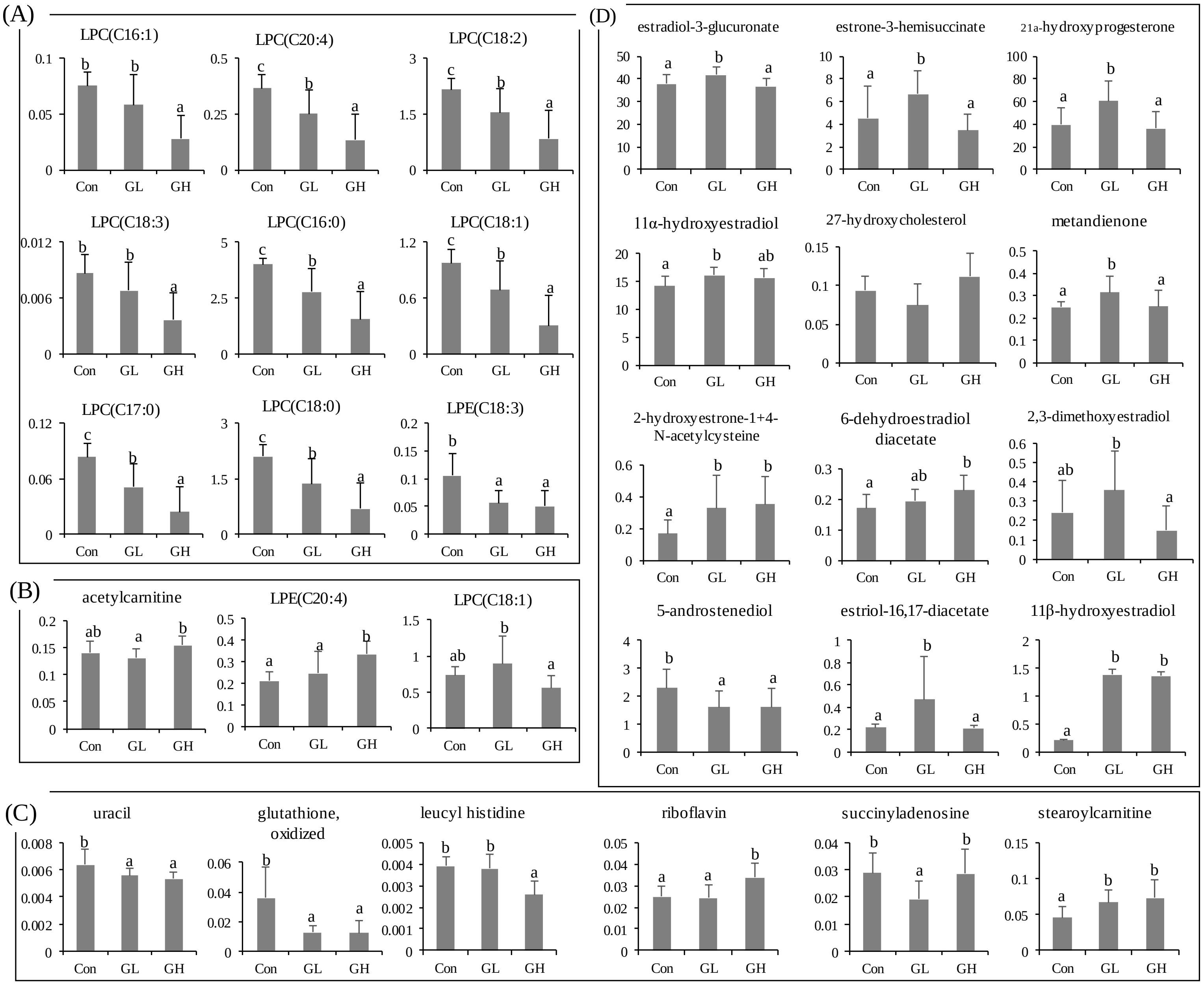

Supplementary Figure 4. Relative abundance of identified metabolites from plasma (A), liver (B), and kidney (C) metabolites and steroid hormones (D) from rats treated with oral administration of the ginseng extract or water. Different letters on the bars indicate d significant differences at $p < 0.05$.

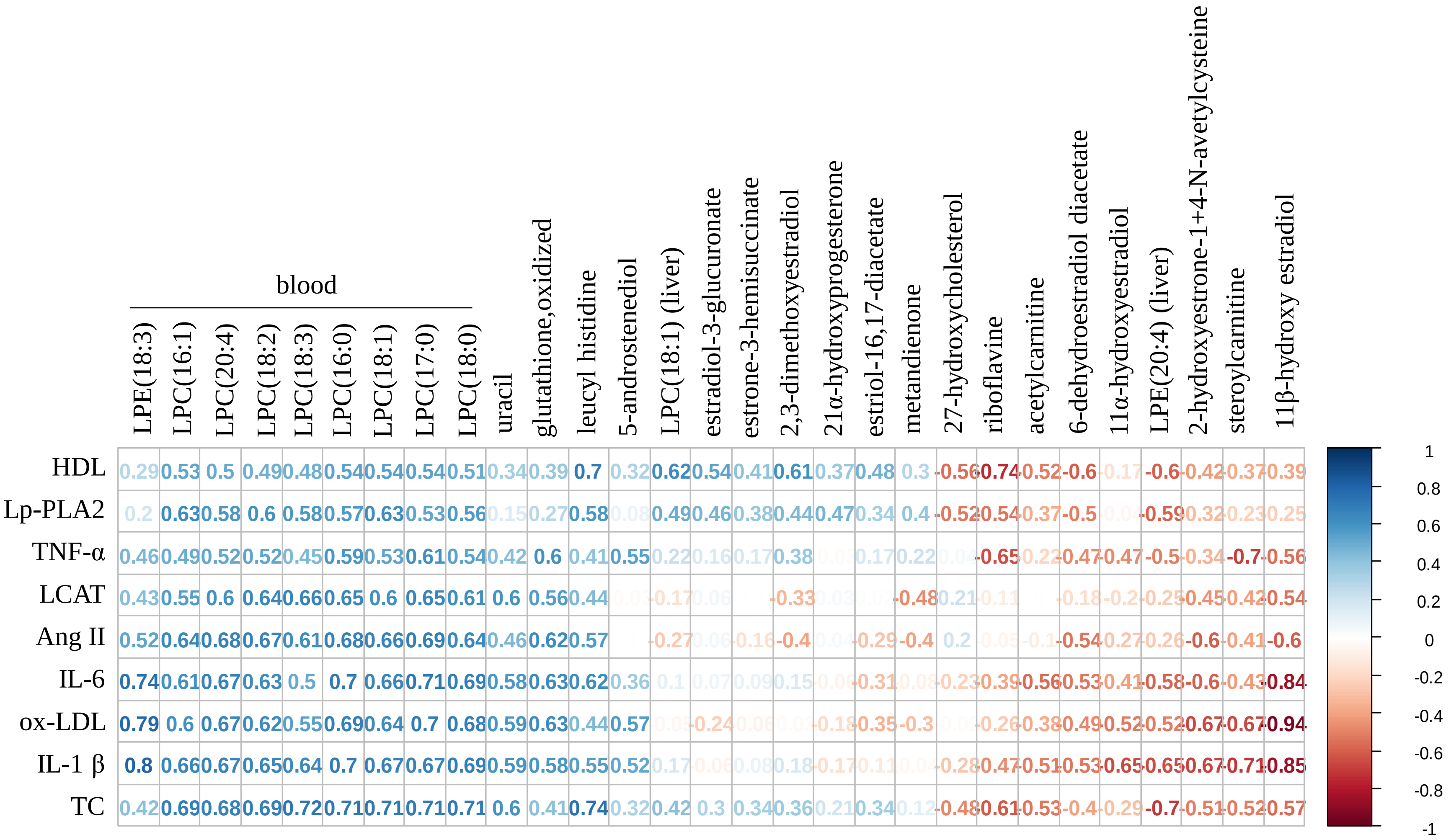

Supplementary Figure 5. Pearson correlation coefficients of the relationship between identified metabolites and steroid hormones and blood health-related factors. The correlation matrix was analyzed and visualized with a heat map generated with the R corrplot package. Positive correlations are shown in blue, and negative correlations are shown in red.
